# Women's Health in Rugby: It Is More Than Orthopedics

**DOI:** 10.7759/cureus.104767

**Published:** 2026-03-06

**Authors:** Stephanie M Pannell, Thomas J Papadimos

**Affiliations:** 1 Surgery, College of Medicine and Life Sciences, The University of Toledo, Toledo, USA; 2 Anesthesiology, Surgery, and Critical Care, College of Medicine and Life Sciences, The University of Toledo, Toledo, USA

**Keywords:** health surveillance, rugby, sports-related injuries, women and health, women-centered care

## Abstract

Rugby is a globally expanding sport for women, yet research on female-specific health and safety issues remains limited. While orthopedic injuries are the most common and are a concern, the differences in physiology, anatomy, and social context between men and women call for a broader and more gender-informed approach to player health, safety, and welfare. There is a need for gender-specific injury surveillance, which includes not only orthopedic injuries, but also breast injuries, pelvic floor dysfunction, and postpartum effects that impair playing. Strengthening research funding and integrating sex-specific findings into practice are essential to ensure safe, equitable participation and optimal performance for women rugby players.

## Editorial

Rugby is a very physically demanding sport played by men and women. While both are subjected to the rigors of the sport, there are significant differences and concerns regarding the physiology, anatomy, and social context between the sexes. Women’s rugby is undergoing an explosive expansion worldwide [1]. Nearly three million women play rugby across the globe, yet research into how the sport particularly affects women is lacking and requires further development and funding. Two years ago, an in-depth review of the needs of women rugby players was addressed in the medical literature [2-7]. Here, we highlight our concerns regarding women’s rugby to the international medical and sports communities regarding safe play during matches and practice in support of the sport. Especially, as it relates to injuries of the head (concussion) and neck, joints, menstrual and hormonal contraceptive (HC) considerations, breast health, pelvic floor dysfunction (PFD), rehabilitation in the postpartum period, the need for appropriate training facilities and time, and the need for robust injury surveillance systems.

The first thing that may come to mind in such a contact sport is orthopedic injuries. And, of course, it is a major concern. However, women differ from men in this respect. Trainers must realize that women have a neck isometric strength that is 47% less than that of male players, along with a smaller neck girth and sternocleidomastoid physiological cross-sectional area (PCSA) [8,9]. It has been demonstrated that an uncontrolled whiplash in university union rugby players occurs 100 times more in women than men (>50% vs. 0.5%) [8]. Furthermore, this report also demonstrated that women’s heads contacted the ground during play more frequently than men (26% vs. 10%) [8]. Whiplash injury that occurs in women is highly consequential because the cervical facet joints (C4-C5) respond differently during the event than in the normal physiology of extension [10,11]. This puts the joint capsule in a more vulnerable situation and position. Therein, increasing the anatomic threat to women rugby players. This finding should influence training protocols, screening, and protective strategies through the provision of sex-specific training methods (how to tackle to protect areas of anatomic vulnerability as compared to men), screening players during the training process for injuries (breasts) and physiologic changes (effects of menstruation), and protective strategies through teaching appropriate tackling methods (to protect breasts and neck, for example) and joint/limb specific strength training. In essence, women use different strategies to stabilize their heads [9]. These mechanics also affect concussive injury. It has been demonstrated that women rely more on neuromuscular activation to steady their head, whereas men use their increased neck girth, sternocleidomastoid PCSA, and flexion strength. Also, the baseline electromyography (EMG) differs between the sexes, as does the muscle onset latency [9]. When examining the role of concussion history and sex of the adolescent players, it is also of interest that higher concussive scoring occurred in those who had previous concussions across both sexes, but women had higher scores than men when evaluated for “cognitive/fatigue, ocular and sleep profiles, concussion symptoms, anxiety, and depression” [12]. This study was done in adolescent rugby players (n = 149, 63 females). In both sexes, higher scores reflected a history of concussion, a higher concussion symptom severity, and more depression. Female players had significantly higher scores across cognitive/fatigue, ocular, and sleep clinical profiles, concussive symptoms, anxiety, and depression than males. These observations indicate that when it comes to head and neck injuries, there are differences between the sexes, and such differences should be identified in surveillance systems, training and tackling style, and recovery programs [13-15].

Joints and their related injuries are another area in which there are differences between genders. Hand fractures among male rugby players are secondary to axial loading, whereas in females, crush injuries or hyperextension are the main etiologies [16]. Women rugby players also have a reactive strength index (RSI) and dynamic knee valgus (DKV) angle that differs from their male counterparts, thereby making an argument that protecting women players from knee injury will require targeted programming of ensuring a proper range of motion exercise program, improving biomechanics during jumps and landings, and proper control of posture on such landings to improve the RSI and DKV [17]. There is also a medical argument that women rugby players, and female athletes in general, are at higher risk of shoulder injury because of multidirectional instability (MDI) [18]. This is secondary to varied muscle development and an increased ligamentous laxity [19-21]. This is probably because women have higher levels of serum relaxin and a higher number of relaxin receptors on their ligaments, leading to increased laxity of the shoulder [19].

Menstrual cycles can also play a role in injuries, as well as cause general discomfort. In a recent survey of professional women rugby players in the UK (300 participated out of a pool of 480 players), nearly 52% reported that their menstrual cycle caused them trouble concentrating, and the authors state that “ovarian hormones influence the speed of decision-making and cerebral function, increasing the risk of accidents” [22]. Furthermore, studies have demonstrated that the ligamentous injury rate and joint instability are associated with the late follicular phase (LFP) and with high estrogen concentrations (during the LFP), and are areas of evolving research [23-25]. It is difficult to address joint laxity and injury during the menstrual cycle without addressing HC use and its potential relationship to injury. A survey of 1596 women rugby players from 62 countries found that 606 players (38%) from 33 countries used synthetic HCs [26]. Of the HC-using patients, 25% reported stomach cramps and 22% needed medication to manage symptoms, and 67/606 (11%) self-reported that their performance was impaired [26]. The effects of the menstrual cycle and HC use in women’s rugby require further inquiry.

Breast health and protection are understudied topics, as well as an underreported area of injury; 58% of women playing contact sports experience breast injuries, and 62% of all rugby codes (union, league, and sevens) report such injuries [6,27]. Two types of breast injuries are highlighted in the literature: exercise-induced chest pain (EIBP) and contact breast injury (CBI) [28,29]. EIBP occurs when there is excessive movement of the breast and/or frictional movement during practice or match play, and CBI occurs with blunt force impact [30,31]. These injuries usually occur when being tackled or tackling. Educational sessions on the importance of reporting and the potential consequences of the injury, along with the necessary treatment, are of paramount importance, as are training sessions in how to tackle and what to do when tackled. The simple act of making sure players have appropriately fitting bras can be extremely helpful [6]. It is of great concern that many women in rugby (and other sports) fail to report breast injuries and generally do not receive player education as to the necessity of such reporting [6,27,29,32]. Only about 10% of women in sport seek treatment for injured breasts [33,34]. Medical follow-up is important because conditions such as fat necrosis can occur, which can be misdiagnosed as breast cancer, mastitis, or abscess [35]. It must be noted that women players avoid reporting their breast injury to their coaches and physicians for fear of not being allowed to play; in effect, such injuries can be career-ending [36]. Additionally, women would rather report their difficulties to a female than a male [36].

PFD is another anatomic aspect of playing rugby that women must confront [5,37,38]. Sprinting, jumping, and landing during match play or practice increase intra-abdominal pressure and ground reaction forces, which impact the pelvic floor. A recent survey of elite women’s rugby players reported that half (57%) had pelvic floor symptoms, which included urinary incontinence (41%), anal incontinence (wind or fecal matter) (29%), bladder urgency/frequency (14%), and pelvic pain (12%). Over half of the players (53%) claimed it affected their performance, 34% claimed it affected their training, 25% reported it caused them to avoid certain locomotor activities on the pitch, and 10% claimed it affected their game day focus [37]. Of further note, pregnancy and childbirth, through trauma and an increased pressure load on the pelvic architecture (muscles and passive structures, such as ligaments), can lead to urinary stress incontinence in the perinatal period [4,37]. These changes will be accompanied by the deconditioning that all female athletes go through in the postpartum period [4,39]. It is of interest that several studies have indicated that nulliparous women may have a similar prevalence of incontinence as the peripartum/postpartum players [37,40]. The reason for this similarity has yet to be identified. However, to hypothesize, this sport is an extremely high-impact sport with repetitive collisions, which cause pelvic floor overload and muscle/structural fatigue. Pregnancy and childbirth are obvious risk factors, but even in nulliparous women, the excessive downward force can injure or weaken a healthy pelvic floor (through intra-abdominal pressure and ground reaction forces). Possibly complicating this is the intensity of training, the high volume of training, and the position played (forwards may be at higher risk).

Proper conditioning in the postpartum period is crucial to the successful return of a player, and the team’s coaching and medical staff should be keenly aware of what is required [5]. While women can experience pelvic floor disorders at any time in their life cycle, pregnancy and childbirth are among the most prevalent risk factors because of the load changes they present to the pelvic floor [5]. These changes are not fully understood [41], and “evidence regarding appropriate timeframes to return to sport postpartum is lacking” [5]. A sport-specific tool for PFD has been developed and may be of help to players, coaches, and medical staff [42]. Appropriate staff and player awareness of PFD is extremely important. Education and surveillance of PFD should be incorporated into every organization.

Finally, a suitable rugby environment for women is important. Recent work has emphasized the need for women in managerial positions to create changes of consequence [1,43]. This must occur because women currently have less access to resources, when compared to men, such as facilities, health education, pitch time, education in tackling skills with gender-specific coaching practices, and protection of player welfare, all of which affect player health and performance [1,2,14,43]. And such an environment must also be accompanied by the appropriate cultural, religious, geographical, and biosocial skills required for team effectiveness [7,44,45].

Female domains in rugby differ from those of men. The fact that three million women will be playing rugby on the global stage means that a concerted effort must be made to advance gender-specific injury and player health surveillance systems, mechanisms to react and incorporate the findings of such surveillance systems into everyday practice and education, and increase access to facilities, research funding, and general support. Specific stakeholders can help improve the position of women in rugby (vis-à-vis men), such as governing bodies, coaches, team physicians, and athletic trainers, who can be brought into the mix of those who may contribute to solutions to the aforementioned concerns. First, bring together the stakeholders mentioned above on a regional level to determine what are the primary concerns/threats to women’s health in rugby (starting on the national level may be difficult). Second, once a concern is addressed, then incorporate it into an epidemiologic surveillance system that tracks the injuries alluded to above or those determined by the stakeholder group. Third, seek funding to store and evaluate the epidemiologic data. Fourth, act on the results of the surveillance system(s). Such an effort is important to the health of women who play rugby.
